# The STRIPAK Complex Regulates Response to Chemotherapy Through p21 and p27

**DOI:** 10.3389/fcell.2020.00146

**Published:** 2020-03-17

**Authors:** Carmen Rodriguez-Cupello, Monica Dam, Laura Serini, Shan Wang, David Lindgren, Emelie Englund, Pontus Kjellman, Håkan Axelson, Alberto García-Mariscal, Chris D. Madsen

**Affiliations:** Division of Translational Cancer Research, Department of Laboratory Medicine, Lund University, Lund, Sweden

**Keywords:** breast cancer, STRIPAK, cell cycle, p21, p27, DNA damage response, chemotherapy

## Abstract

The STRIPAK complex has been linked to a variety of biological processes taking place during embryogenesis and development, but its role in cancer has only just started to be defined. Here, we expand on previous work indicating a role for the scaffolding protein STRIP1 in cancer cell migration and metastasis. We show that cell cycle arrest and decreased proliferation are seen upon loss of STRIP1 in MDA-MB-231 cells due to the induction of cyclin dependent kinase inhibitors, including p21 and p27. We demonstrate that p21 and p27 induction is observed in a subpopulation of cells having low DNA damage response and that the p21^high^/γH2AX^low^ ratio within single cells can be rescued by depleting MST3&4 kinases. While the loss of STRIP1 decreases cell proliferation and tumor growth, cells treated with low dosage of chemotherapeutics *in vitro* paradoxically escape therapy-induced senescence and begin to proliferate after recovery. This corroborates with already known research on the dual role of p21 and indicates that STRIP1 also plays a contradictory role in breast cancer, suppressing tumor growth, but once treated with chemotherapeutics, allowing for possible recurrence and decreased patient survival.

## Introduction

Over the past few years, extensive functional and mechanistic research has been conducted to resolve the framework of the Striatin Interacting Phosphatase and Kinase (STRIPAK) complex. The accumulated findings have linked specific components of the complex to various biological functions including vesicular trafficking (Zhang et al., 2013; Lant et al., 2015), Golgi assembly (Kean et al., 2011), Hippo signaling (Ribeiro et al., 2010; Zheng et al., 2017), autophagy (Huang et al., 2017), cell migration (Madsen et al., 2015; Bazzi et al., 2017), and cell cycle control (Cornils et al., 2011; Frost et al., 2012; Kazmierczak-Baranska et al., 2015; Pandey et al., 2017). Substantiated by these findings, the STRIPAK complex is supervising embryogenesis and development (Lant et al., 2015; Madsen et al., 2015; Sakuma et al., 2015, 2016; Bazzi et al., 2017; Pal et al., 2017; Zheng et al., 2017), circadian rhythms (Andreazza et al., 2015), type 2 diabetes (Chursa et al., 2017), and progression of cancer (Wong et al., 2014; Zhang et al., 2014; Madsen et al., 2015; Huang et al., 2017).

The STRIPAK complex is an evolutionarily conserved supramolecular complex; holding the PP2A phosphatase in complex with its striatin-family of regulatory subunits (STRN, STRN3, STRN4), the two hippo kinases (MST1/MST2), the three GCKIII kinases (MST3, MST4, SOK1) and various scaffolding proteins (Glatter et al., 2009; Goudreault et al., 2009; Ribeiro et al., 2010; Couzens et al., 2013). It is believed that the scaffolding proteins, including SLMAP, SIKE, STRIP1 (FAM40A), STRIP2 (FAM40B), direct and uphold PP2A/Striatin phosphatase specificity, and loss of these proteins consequently disassemble the STRIPAK complex; leading to hyper-phosphorylation of PP2A/Striatin target proteins. This is, for example, observed upon loss of SLMAP, which induces hyper-phosphorylation of MST1/2 kinases (Bae et al., 2017; Zheng et al., 2017; Tang et al., 2019), while loss of STRIP1 induces hyper-phosphorylation of MST3/4 kinases (Madsen et al., 2015).

The *in vivo* function of STRIP1 has been described in multiple eukaryotic organisms. In the filamentous fungus *Neurospora crassa*, the *Strip1* homolog is important for hyphal fusion (Xiang et al., 2002) and required for normal recovery from pheromone arrest in G1 of the cell cycle (Kemp and Sprague, 2003). In yeast, the *Strip1* homolog connects the Golgi, the centrosome, and the nuclear envelope to organize mitotic progression (Frost et al., 2012). The yeast homolog also antagonizes mTORC2 signaling by promoting dephosphorylation of TORC2 substrates (Pracheil et al., 2012). In *Drosophila melanogaster*, the *Strip1* homolog regulates border cell migration (Madsen et al., 2015), serves as a molecular linker for early endosome organization in axon elongation (Sakuma et al., 2014), and regulates the circadian clock by dephosphorylating the circadian oscillator CLOCK during daytime (Andreazza et al., 2015). The *Strip1* homolog in the fruit fly has also been linked to cell proliferation by antagonizing Hippo signaling and by supporting RAS/MAPK signaling (Ashton-Beaucage et al., 2014). In the mouse embryo, loss of *Strip1* arrests mesoderm migration after the gastrulation epithelial-to-mesenchymal transition (Bazzi et al., 2017). Indeed, STRIP1 has been shown to regulate cytoskeleton dynamics and cell migration on several occasions (Bai et al., 2011; Sakuma et al., 2015, 2016; Suryavanshi et al., 2018). We discovered that the STRIPAK complex is an important and ancient regulator of plasticity of cell migration during both developmental processes and cancer metastasis (Madsen et al., 2015). We demonstrated that loss of STRIP1 induces strong activation of the two MST3&4 kinases, consequently inducing breast cancer cells to metastasize using actomyosin-driven amoeboid migration. These data were the first to demonstrate that perturbation of STRIP1 could affect tumorigenesis in breast cancer (Madsen et al., 2015). In this paper, we continue to elaborate on the molecular and biological functions of STRIP1 and MST3&4 in breast cancer. We show that loss of STRIP1 induces the expression of cyclin dependent kinase inhibitors (CKI) including CDKN1A (p21), which leads to cell cycle arrest and reduced tumor growth. Surprisingly the strong induction of p21 also has an inconvenient effect if cells are treated with chemotherapeutic, as it promotes a proliferative cell fate rather than inducing a senescent phenotype when treated with sub-lethal doses of chemotherapeutics.

## Materials and Methods

### Cell Culturing and Transfections

Human MDA-MB-231 breast cancer cells (ATCC) were cultured in Dulbecco's Modification of Eagle's Medium (DMEM) supplemented with 10% fetal bovine serum and 1% penicillin-streptomycin under 5% CO2 and 37°C. siRNA transfections were performed using Lipofectamine 2000 (ThermoScientific). In brief, cells were subjected to transfection in serum-free OptiMEM using 25 nM siRNA. After 24 h of transfection, the cells were re-plated for subsequent analyses. Seventy-two hours post-transfection, cells were collected for flow cytometry, immunoblotting, or fixed for immunofluorescence. The following siRNAs were used in the study: Hs_FAM40A_2 FlexiTube siRNA (SI00383796, Qiagen), Hs_FAM40A_5 FlexiTube siRNA (SI04198789, Qiagen), Hs_FAM40A_7 FlexiTube siRNA (SI04295949, Qiagen), STRIP1_35 (s39935, ThermoFisher), STRIP1_36 (s39936, ThermoFisher), Hs_FAM40B_7 FlexiTube siRNA (S104300618, Qiagen), siGENOME Human STK24 (MST3) siRNA (D-004872-23, Horizon Discovery), siGENOME Human STK26 (MST4) siRNA (D-003753-04, Horizon Discovery), siGENOME Human STK25 siRNA (D-004873-02, Horizon Discovery), siGENOME Human PDCD10 (CCM3) siRNA (D-004436-01, Horizon Discovery), CDKN1A_01 (s417, ThermoFisher), CDKN1A_02 (s415, ThermoFisher), CDKN1B_01 (s2837, ThermoFisher), and CDKN1B_02 (s2838, ThermoFisher). Treatment with Doxorubicin (Sigma) and Cisplatin (Merck) for high dosage were supplemented into culture media at 1 μM for 6 h, beginning 72 h post transfection. For senescence and recovery with low dosage, doxorubicin and cisplatin were supplemented at 50 nM and 250 nM, respectively, for 24 h, beginning 48 h post-transfection, and allowed to recover in normal media for another 96 h.

### RNA-Sequencing

Total RNA was prepared 72 h post-transfection using RNeasy (Qiagen), according to the manufacturer's instructions. RNA was treated with DNase I on the columns before eluting the RNA. RNA-sequencing was conducted on samples from 3 independent experiments. Quality control of the RNA and RNA-sequencing was performed by The Eukaryotic Single Cell Genomics facility, Lund University. Bioinformatic validation and quantifications were performed in house. GSEA analysis was performed using Broad Institute analysis software and publicly available gene sets. All analyses were run using 1,000 permutations.

The RNA-sequencing data generated in this study have been deposited in the GEO database under accession GSE145618.

### Proliferation Analysis

Proliferation curves of cells were based on cell count analysis after siRNA-transfection, beginning 2 days post-transfection where the gene and protein knockdown was at its maximum. Proliferation with drug usage was performed using ethynyl-20-deoxyuridine (EdU) Proliferation Kit (Abcam) according to manufacturer's protocol. For immunofluorescence staining, a final concentration of 40 μM EdU was supplemented to cells in culture medium for 2 h prior to fixation. In total hundreds to thousands of cells were quantified per siRNA transfection, by assessing 50–100 cells per image, with 5 images per condition, and at least 3 independent repeats. For flow cytometry analysis, a final concentration of 20 μM EdU was supplemented to cell in culture medium for 2 h prior to harvesting. Gating protocol for EdU proliferation analysis was performed according to manufacturer's protocol using approximately 10,000 cells per repeat.

### Immunoblotting

Western blotting was performed according to standard procedures. Cells were washed with ice-cold Dulbecco's phosphate-buffered saline (PBS) and then lysed using 1 × Laemmli buffer with 50 mM DTT and further processed through sonication using Biorupture (Diagenode). The samples were resolved in 4–20% Tris-Glycine gels (Invitrogen) and subsequently transferred to a polyvinylidene difluoride (PVDF) membrane (Amersham) using wet transfer. PVDF membranes were blocked with 5% milk in Tris-buffered saline with 0.02% Tween20 (TBS-T) for 1 h and then probed with primary antibodies diluted in 3% BSA in TBS-T overnight, and subsequently with secondary antibody conjugated to horseradish peroxidase diluted in 5% milk, TBS-T for 1–2 h. The specific proteins were detected with Amersham Imager 600 (GE Healthcare) after incubation with Luminata Crescendo/Forte Western HRP substrate (EMD Millipore). The following antibodies were used for western blotting: FAM40A/STRIP1 (ab199851, Abcam, 1:250), p21 (sc6246, Santa Cruz Biotechnologies, 1:500), p27 (sc1641, Santa Cruz, 1:500), cyclin A (sc271682, Santa Cruz, 1:1000), phospho-RB (8516, Cell Signaling, 1:1000), phospho-LATS1(Ser909) (9157, Cell Signaling, 1:1000), pMST1/2 (3681, Cell Signaling, 1:1000), pGCKIII [Anti-MST4 + MST3 + STK25 (phospho T174 + T178 + T190)] (ab76579, Abcam, 1:1000), phospho-AKT (Ser473) (4060, Cell Signaling, 1:1000), AKT (4691, Cell Signaling, 1:1000), phospho-GSK-3β (5558, Cell Signaling, 1:1000), γH2AX (2577, Cell Signaling, 1:400), tubulin (5335, Cell Signaling, 1:50000), anti-Rabbit HRP (7074, Cell Signaling, 1:2000), and anti-Mouse HRP (7076, Cell Signaling, 1:2000).

### Flow Cytometry for Cell Cycle

Cells were collected by trypsinization and fixed with ice-cold 70% ethanol. After washing, the cell pellet was resuspended in a staining solution of 50 μg/ml propidium iodide (PI) (Sigma) and 100 μg/ ml RNase in PBS. The cell cycle phase of approximately 10,000 cells was determined by FACSverse (BD Biosciences) and further analyses of collected data points was performed using FlowJo. The whole cell population is first gated (R1) according to forward and side scatter. Further gating is performed by measuring the area (PI-A) and the width of the collected PI signal (PI-W) for removal of apoptotic cells and doublets (R2). Single cells are then sorted into subpopulations G0/G1, S, and G2/M, represented in a histogram with PI-A on the x-axis. For nocodazole treatment of cells, 200 nM of nocodazole (Sigma) was added to culture medium 18 h prior to collection.

### Serum Starvation for Inducing Hippo Signaling

Glass bottom culture plates (Mattek) were coated with collagen/matrigel and allowed to polymerize for 1 h prior to addition of cells. Collagen/matrigels were made with 10% FBS, 40% Rat-tail collagen I (Corning), 20% Matrigel Basement Membrane Matrix (Corning), a 5X collagen buffer, and culture media. Cells were plated 24 h post-transfection and incubated for a further 48 h. Prior to fixation, cells were placed in serum-free media for either 30 or 60 min. At least 5 images were taken of each condition.

### Immunofluorescence and Confocal Microscopy

Cells were plated on glass bottom culture plates (Mattek) for confocal microscopy. The cells were PFA-fixed 72 h post-transfection. The cells were permeabilized in 0.2% Triton X-100, PBS and then blocked with 3% BSA, PBS prior to overnight staining with primary antibodies diluted in 1% BSA, PBS. A cocktail of AlexaFluor-conjugated secondary antibodies (ThermoFisher, 1:400) with DAPI (Sigma, 1:500) and Phalloidin-TRITC (Sigma, 1:500) were then added to the samples. All fluorescent images were acquired using a Leica SP8 or a Zeiss LSM710 confocal microscope. Five images per condition were taken, containing 50–100 cells each, for each repeat, with at least 3 independent repeats. The following antibodies were used: YAP (sc101199, Santa Cruz Biotechnologies, 1:100), p21 (sc6246, Santa Cruz Biotechnologies, 1:50), p27 (3686, Cell Signaling, 1:800), and γH2AX (2577, Cell Signaling, 1:800).

### Generation of Stable Crispr/Cas9 Knockout Cell Lines

The lentiCRISPRv2 (Gecko, Addgene) was used as described previously (Garcia-Mariscal et al., 2018). Single-guide RNAs (sgRNAs) targeting genes of interest coding regions were designed and cloned into the lentiviral vector lentiCRISPRv2 (Gecko, Addgene) according to the manufacturer's instructions. All sgRNAs used were selected for low off-target efficiency using algorithms at crispor.tefor.net. The oligonucleotide sequences corresponding to the sgRNAs were: STRIP1 sgRNA#1: F: 5′-CACCGCTGGTTGCGGTTGAACTCGC-3′, R: 5′-AAACGCGAGTTCAACCGCAACCAGC-3′: STRIP1 sgRNA#2: F: 5′-CACCGTGTTTGTTGTTCACGATCAG-3′, R: 5′- AAACCTGATCGTGAACAACAAACAC-3′; STRIP1 sgRNA#3: F: 5′- CACCGAGCCGCACAGCCACCACCCG-3′, R: 5′-AAACCGGGTGGTGGCTGTGCGGCTC-3′; STRIP1 sgRNA#4: F: 5′-CACCGCTATTCGGAGTCACCAGACC-3′, R: 5′-AAACGGTCTGGTGACTCCGAATAGC-3′. HEK293T cells were used for the lentivirus production by transfection of lentiCRISPRv2 vector together with pCMV-VSV-G and psPAX2 using Lipofectamine 2000 according to the manufacturer's instructions. Next day, HEK medium was exchanged and after 24 h, the supernatant containing the viral particles were collected, mixed (1:1) with fresh medium containing 8 μg/ml polybrene (Sigma Aldrich), and added to the MDA-MB-231 cells. The MDA-MB-231 cells were infected with lentivirus for 24 h, before exchanging with fresh medium containing 1 μg/ml puromycin for 5 days. The gene-modified cells were not purified further, and therefore used as a pool for the subsequent experiments. All four sgRNAs were validated and sgSTRIP1#3 was chosen for the animal experiments.

### Animal Experiments

All experiments were carried out according to institutional guidelines and approved by the local ethics committee in Lund, permit number 12562/2018. Female NSG mice were purchased from Jackson. Mice (8 weeks old) were orthotopically injected into the 4th inguinal mammary fat pad with 1 × 10^6^ MDA-MB-231-CRISPR^CONTROL^ cells on the left side and 1 × 10^6^ MDA-MB-231-CRISPR^STRIP1^ cells on the right side. Tumors were removed 17 days post-injection. The tumor volume was calculated accordingly: volume = width^2^ × length × 0.52.

### Image Analysis

Quantification of western blots and immunofluorescence images was performed using ImageJ/Fiji software (imagej.net/Fiji). At least 5 images were taken for each condition for at least 3 repeats. Cell number was obtained through DAPI staining and software calculation by creation of regions of interest (ROIs) for each nucleus, with a 50-pixel exclusion. Nuclear ROIs were used to obtain mean nuclear staining of p21 and γH2AX. In house macros for Fiji software were created to unbiasedly extract intensity values of staining for images.

### Statistical Analysis

All graphs and statistical tests were created using GraphPad Prism. All graphs are depicted as mean ± SD. Statistical tests were performed using one-way or two-way ANOVA or unpaired student's *t*-test (two-tailed). All tests were performed at least three independent times. *P < 0.05, **P < 0.01, ***P < 0.001, ****P < 0.0001.

## Results

### Loss of STRIP1 Arrests Cells in the G1-Phase of the Cell Cycle

**Figure 1:**
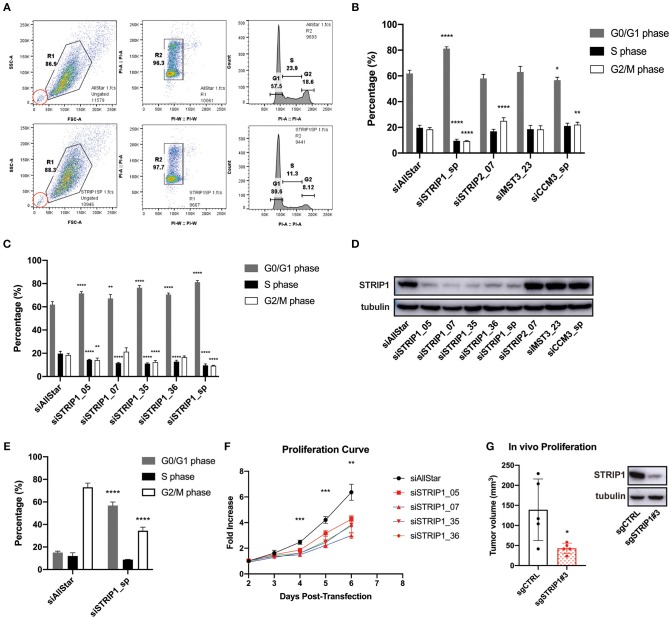
Loss of STRIP1 arrests cells in the G1-phase of the cell cycle. **(A)** Gating strategy of cell cycle analysis using flow cytometry of control/AllStar (top) and STRIP1 depleted (bottom) cells. MDA-MB-231 cells were stained with propidium iodide (PI) to analyze the DNA content. Side scatter area (SSC-A) describes the granularity of each passing cell, forward scatter area (FSC-A) measures the cell size. A population of cells (R1) is gated (left) and the cells are plotted according to the area and width of detected PI (middle). Three subpopulations (R2) describe the amount of DNA content (G0/G1, S, G2/M) and can be better visualized in a histogram (right). Inset, in red, indicates unchanged quantity of apoptotic cells between control and STRIP1 depleted cells. **(B)** Cell cycle analysis of MDA-MB-231 cells siRNA-depleted for core components of the STRIPAK complex incl. STRIP1, STRIP2, MST3, and CCM3. Depletion of STRIP1 leads to a greater percentage of cells in the G0/G1 phase compared to control. A smart pool (sp) of differently targeted siRNAs were used for STRIP1 and CCM3. **(C)** Cell cycle analysis of multiple STRIP1 siRNAs and combined smart pool (sp). The use of individual siRNAs for STRIP1 similarly result in a higher percentage of cells in G0/G1 phase compared to control. **(D)** STRIP1 protein knockdown efficiency of siRNA-depleted MDA-MB-231 cells was demonstrated by immunoblotting. **(E)** Cell cycle analysis of control and STRIP1 depleted MDA-MB-231 cells after treatment with nocodazole for 18 h to synchronize cells in G2/M phase. Loss of STRIP1 maintains the cells at a G0/G1 arrest. All cell cycle analyses were conducted at least three independent times. **(F)** Cell proliferation assay of STRIP1 siRNA-depleted MDA-MB-231 cells. Proliferation analysis was performed at least five independent times. Statistical analysis using two-way ANOVA was performed on siSTRIP1_05 compared to siAllStar, **P < 0.0021 and ***P < 0.0002. **(G)** MDA-MB231 cells were genetically modified using CRISPR/Cas9 for removal of STRIP1 and orthotopically injected into the mammary fat pad of NSG mice. Tumor volume quantification shows decreased size with loss of STRIP1. Results obtained are from five individual animals carrying one tumor of each. Knockout efficiency of CRISPR cells was demonstrated by immunoblotting. All statistical tests of the cell cycle analyses were performed using one-way ANOVA, *P < 0.05, **P < 0.01, ***P < 0.001, ****P < 0.0001 to siAllStar.

We previously demonstrated that the STRIPAK complex is an important regulator of breast cancer cell migration and metastasis in mouse models (Madsen et al., 2015). Interestingly, previous findings also suggest that the STRIPAK complex may, as well, perturb the cell cycle. We therefore decided to investigate if loss of individual STRIPAK components would interfere with the cell cycle. A flow cytometry cell cycle analysis of MDA-MB-231 breast cancer cells was conducted 72 h after siRNA-depletion of individual STRIPAK genes known to regulate cancer cell migration and metastasis. The siRNA-mediated gene-depletion was conducted with already validated siRNAs (Madsen et al., 2015) and demonstrated that loss of STRIP1 significantly increased the numbers of cells arrested in the G0/G1 phase of the cell cycle, while lowering the numbers of cells in S and G2/M, as compared to the siRNA control (siAllstar) cells (Figures 1A,B). The siRNA-depletion of STRIP2 and CCM3 induced minor differences, while loss of MST3 did not affect the cell cycle, probably due to redundancy from MST4 (Madsen et al., 2015). We decided to focus on the role of STRIP1 due to its strong impact. As the flow analysis was conducted using a smart pool of STRIP1 siRNAs, we confirmed our findings using four individual siRNAs, obtained from different companies (Figure 1C). Immunoblotting analysis validated that all siRNAs successfully depleted the STRIP1 protein (Figure 1D).

We next set out to investigate whether the cells were able to progress through the cell cycle by treating them with nocodazole, an agent that interferes with the polymerization of microtubules. Thus, adding nocodazole to proliferating cells will arrest them in G2/M phase due to the spindle assembly checkpoint. siRNA-depleted MDA-MB-231 cells were treated with 200 nM nocodazole for 18 h before collecting them for flow cytometry. Approximately 70% of control cells were found to be in G2/M phase after nocodazole treatment (Figure 1E). On the contrary, close to 60% of STRIP1-depleted cells were still found in the G0/G1 phase, emphasizing the role of STRIP1 in the G1-exit of the cell cycle (Figure 1E). We then tested if loss of STRIP1 affected overall cell proliferation. Our data shows that loss of STRIP1 slightly reduced proliferation rate (Figure 1F), while the numbers of apoptotic cells were unchanged, according to the flow cytometry analysis (Figure 1A, inset). These data demonstrate that loss of STRIP1 maintain or prolong cells in the G1-phase and, as a consequence, lower the net proliferation of MDA-MB-231 cells. To substantiate these findings, we genetically manipulated MDA-MB-231 cell using CRISPR/Cas9 technology. The knockdown efficiency was validated, and MDA-MB-231-CRISPR^CONTROL^ and -CRISPR^STRIP1^ cells were implanted orthotopically into the mammary fat pad of NSG mice and the tumor size quantified (Figure 1G). These findings demonstrate that loss of STRIP1 reduces cell proliferation and tumor growth.

### Loss of STRIP1 Induces Expression of CDK Inhibitors p21 and p27

Cells in the G1 phase are preparing to enter the S-phase, but must ensure that the genome is undamaged and that there are enough resources to replicate the DNA (Bertoli et al., 2013). The G1 checkpoint is regulated by cyclin-dependent kinase (CDK) inhibitors that physically interact and inhibit the activity of CDKs, thus preventing the cells from entering the cell cycle prematurely (Bertoli et al., 2013). Cyclins are proteins that control cell cycle progression by activating CDKs. In early G1 phase, CDK4/6 interacts with cyclin D to mono-phosphorylate Retinoblastoma (RB). The phosphorylation of RB is further enhanced by the cyclin E/CDK2 complex, and as RB gradually becomes more phosphorylated throughout the G1 phase, it dissociates from the transcription factor E2F, allowing E2F to drive the expression of genes needed to enter the S phase and for the initiation of DNA replication (Bracken et al., 2004; Bertoli et al., 2013).

**Figure 2:**
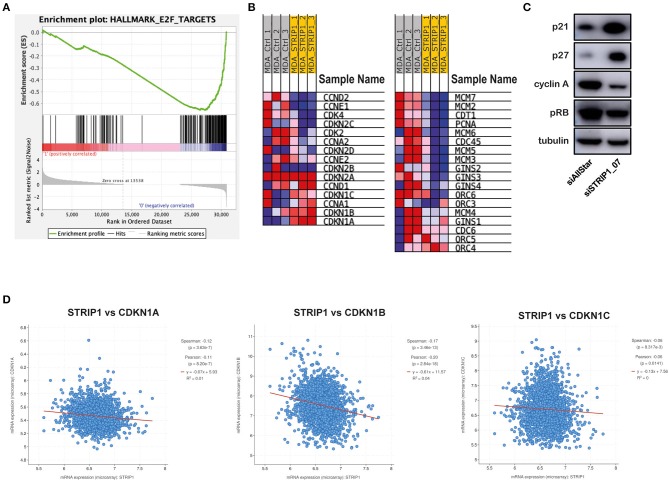
Loss of STRIP1 induces expression of CDK inhibitors p21 and p27. **(A)** Gene Set Enrichment Analysis of RNA-sequencing data of siRNA-depleted MDA-MB-231 cells. The data shows an overall reduction in E2F targeted genes after loss of STRIP1 in MDA-MB-231 cells. **(B)** Heat map of selected genes involved in the regulation of cell cycle progression in control and STRIP1 siRNA-depleted cells. **(C)** Immunoblotting analysis of cell cycle regulating proteins p21, p27, Cyclin-A, pRB. Expression of p21 and p27 are enhanced upon STRIP1 loss while Cyclin-A and pRB are depleted upon STRIP1 loss. All immunoblotting analyses were conducted at least three independent times. **(D)** Dataset analysis showing negative correlation of STRIP1 to CDK inhibitors CDKN1A, CDKN1B, and CDKN1C.

We performed RNA sequencing of siRNA-depleted MDA-MB-231 cells to look for cell cycle regulated changes. Gene set enrichment analysis (GSEA) of STRIP1-depleted cells demonstrated a general decrease in E2F-target genes responsible for the G1/S transition (Figure 2A). The analysis identified CCND2 (cyclin D2), CCNE1 (cyclin E1), as well as CDK2 and CDK4 to be downregulated after loss of STRIP1 (Figure 2B). On the contrary, the expression of all members of the CIP/Kip family of CDK inhibitors; CDKN1A (p21), CDKN1B (p27), and CDKN1C (p57), were all increased, while the INK4 family of CDKs inhibitors; CDKN2A (p16), CDKN2B (p15), CDKN2C (p18), and CDKN2D (p19) did not change after loss of STRIP1 (Figure 2B). The RNA sequencing analysis also demonstrated that genes encoding the functional DNA helicase machinery, responsible for unwinding the DNA template at the replication fork (Leman and Noguchi, 2013), were all downregulated after loss of STRIP1. These genes included the CDC45, the mini-chromosome maintenance (MCM2-7) proteins, and the go-ichi-ni-san (GINS) complex, as well as CDT1 and CDC6; two proteins responsible for the recruitment of the MCM complex to the replication origins (Figure 2B). Immunoblotting analyses confirmed that the levels of p21 and p27 were augmented upon loss of STRIP1, while the levels of cyclin A and phosphorylated RB were reduced (Figure 2C). These findings are in accordance with what is expected of cells arrested in the G1 phase.

To justify our cell culture experiments, we took advantage of publicly available datasets from breast cancer patients enrolled in the METABRIC [Molecular Taxonomy of Breast Cancer International Consortium] cohort (Curtis et al., 2012; Pereira et al., 2016). The clinical data supported our findings and demonstrated a significant inverse correlation between expression of STRIP1 and the three members of the CIP/Kip family of CDK inhibitors; CDKN1A (p21), CDKN1B (p27) and CDKN1C (p57) (Figure 2D). Interestingly, the clinical data also revealed an inverse correlation between STRIP1 and members of the INK4 family of CDK inhibitors; CDKN2A, CDKN2B, CDKN2C, and CDKN2D which the cell culture experiment could not recognize (Supplementary Figures 1A–D). Taken together these findings indicate that the level of STRIP1 may regulate the expression of CDK inhibitors independently of induced DNA damage and activation of p53, as MDA-MB-231 cells only express mutant p53.

### p21 and p27 Induction Is Regulated by the MST3 and 4 Kinases

**Figure 3:**
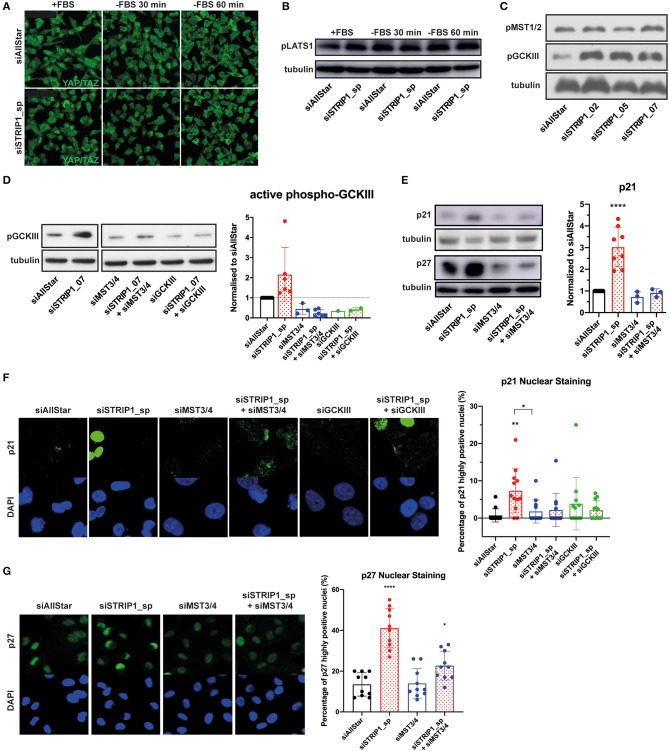
p21 and p27 induction is regulated by activated MST3&4 kinases. **(A–C)** Depletion of STRIP1 in MDA-MB-231 cells does not affect Hippo signaling and YAP/TAZ localization. **(A)** Immunofluorescence analysis of YAP/TAZ localization after STRIP1 depletion in normal media conditions and serum starvation for 30 and 60 min. **(B)** Immunoblotting analysis of phospho-S909-LATS1 under normal media conditions and serum starvation for 30 and 60 min. **(C)** Immunoblotting analysis demonstrates that loss of STRIP1 does not induce phosphorylation of MST1/2. On the contrary, loss of STRIP1 induces strong phosphorylation of the three kinases MST3, MST4, and SOK1 forming the Germinal Center Kinase III (GCKIII) complex. **(D)** Loss of STRIP1 induces auto-phosphorylation of activation loop of the GCKIII complex, as demonstrated by rescuing the effect using kinase specific siRNAs. Included quantification of activated phospho-GCKIII in siRNA-depleted MDA-MB-231 cells. The data shows that loss of STRIP1 induces activation of GCKIII in a MST3&4 dependent manner. **(E)** Immunoblotting analysis demonstrates that siRNA depletion of MST3&4 represses p21 and p27 induction after loss of STRIP1. Included quantification of p21 levels. **(F,G)** Immunofluorescence analysis demonstrates that siRNA depletion of MST3&4 represses p21 and p27 induction after loss of STRIP1. The percentage of MDA-MB-231 cells expressing high levels of nuclear p21 and p27 was quantified. All immunoblotting and immunofluorescence analyses were conducted at least three independent times. All statistical tests were performed using one-way ANOVA, *P < 0.05, **P < 0.01, ****P < 0.0001 to siAllStar unless indicated.

The molecular function of STRIP1 is to maintain close proximity between the PP2A/striatin phosphatase and its targeting substrates. These include the STRIPAK associated kinases: MST1 and MST2 (the two hippo kinases), and MST3, MST4, and SOK1 (the three GCKIII kinases). When STRIP1 is lost, the STRIPAK complex disassembles and the kinases are no longer dephosphorylated and as a result become hyper-activated (Madsen et al., 2015; Tang et al., 2019). Depletion of STRIP1 has previously been linked to Hippo signaling and reduced cell growth. However, siRNA-depletion of STRIP1 in MDA-MB-231 cells did not alter the nuclear localization of the Hippo-controlled YAP/TAZ transcriptional regulators (Figure 3A). The nuclear localization of YAP/TAZ also did not vary in STRIP1-depleted cells under stress conditions of serum starvation (activating the Hippo kinases) when compared to control cells (Figures 3A,B). To reinforce this, we used three different siRNAs targeting STRIP1 and conclusively showed that loss of STRIP1 does not induce phosphorylation of MST1/2 but on the contrary, a strong phosphorylation of GCKIII kinases (Figure 3C). These findings suggest that loss of STRIP1 is not reducing cell proliferation in MDA-MB-231 cells through altered Hippo-signaling. In our previous work, we demonstrated that STRIP1 is a negative regulator of MST3 and MST4 in cancer cells (Madsen et al., 2015). Indeed, loss of STRIP1 induces auto-phosphorylation and activation of the GCKIII kinases, although the importance of SOK1 seem to be minor in MDA-MB-231 cells (Figure 3D). We therefore hypothesized that hyper-activated MST3&4 may be responsible for the induction of p21 and p27 seen after the loss of STRIP1. Indeed, depletion of MST3&4 completely reverted the induced p21 and p27 expression seen after loss of STRIP1 in MDA-MB-231 cells (Figure 3E).

AKT plays an important role in regulating cell cycle progression by phosphorylating p21, thereby reducing its interaction with CDK2/4 and PCNA thus promoting the cells to enter S-phase, and by phosphorylating glycogen synthase kinase-3 (GSK3), which hinders the degradation of β-catenin and as a result enhances cell proliferation (Rossig et al., 2001; Child and Mann, 2006; Karimian et al., 2016). Loss of STRIP1 significantly decreased phospho-AKT and phospho-GSK3β in a MST3&4 dependent manner (Supplementary Figure 1E), suggesting that MST3&4 kinases may contribute to the cell cycle regulation by influencing AKT-signaling.

### Active MST3 and 4 Kinases Produce a Subpopulation of Cells Expressing High Levels of p21 and p27

It has been demonstrated that p21 expression can be heterogeneous in an isogenic population of cells (Overton et al., 2014). We therefore decided to examine p21 expression in single cells by immunofluorescence analysis. To our surprise, only a sub-population of MDA-MB-231 cells demonstrated strong nuclear p21 staining after loss of STRIP1 (Figure 3F). The quantitative analysis demonstrated that loss of STRIP1 induced high levels of p21 in around 10% of the cells, as compared to control cells having <1% of cells expressing high levels of p21 (Figure 3F). Importantly, co-depletion of MST3&4 almost entirely reverted the numbers of p21^high^-expressing cells to levels comparable to control cells (Figure 3F). We then asked ourselves if MST3&4 regulated p27 in a similar way. Indeed, loss of STRIP1 induces high p27 expression in a subpopulation of the cells (Figure 3G). These data support a scenario where the loss of STRIP1, and the subsequent activation of MST3&4, can create a heterogeneous subpopulation of cells expressing high levels of p21 and p27 within an isogenic population of breast cancer cells.

### STRIP1 Regulates DNA Damage Response

**Figure 4:**
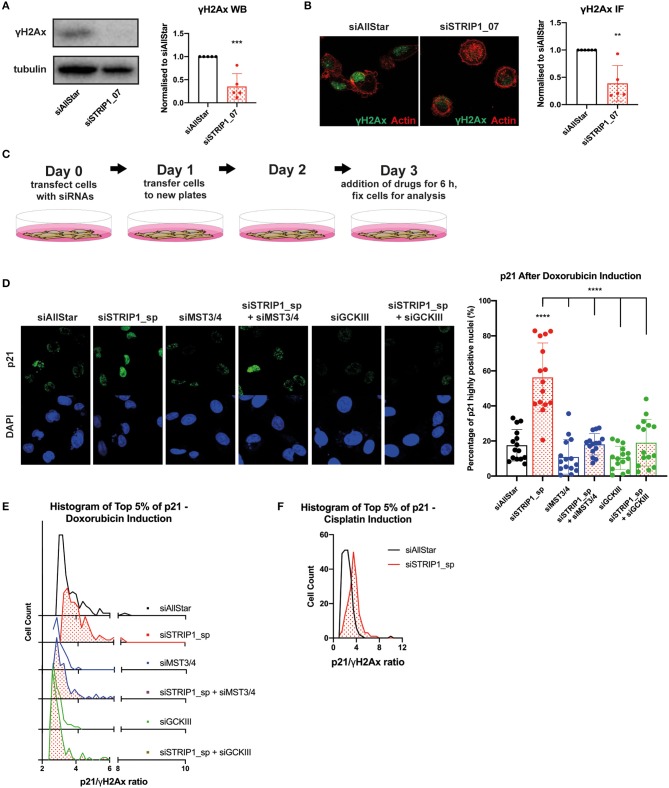
STRIP1 regulates DNA damage response. **(A)** Loss of STRIP1 suppresses the DNA damage response as measured by γH2AX immunoblotting with quantification. **(B)** Loss of STRIP1 suppresses the DNA damage response also measured by immunofluorescence with quantification. **(C)** Experimental set-up of the drug treatment experiment. **(D–F)** siRNA-depleted MDA-MB-231 cells were treated with high dose chemotherapeutics 72 h post-transfection. **(D)** Depletion of MST3&4 represses p21 induction after loss of STRIP1 even after high dose chemotherapeutics. The percentage of siRNA-depleted MDA-MB-231 cells expressing high levels of nuclear p21 after high dose doxorubicin treatment was quantified. **(E)** Loss of STRIP1 increases the p21/γH2AX ratio in a MST3&4 dependent manner after high dose chemotherapy, indicated by the shift toward a higher ratio. Cells investigated are at the top 5% intensity of nuclear p21 using immunofluorescence analysis. **(F)** Increased p21/γH2Ax ratio is also seen in STRIP1 depleted cells after high dose treatment with another chemotherapeutic, cisplatin. Cells investigated are at the top 5% intensity of nuclear p21. All immunoblotting and immunofluorescence analyses were conducted at least three independent times. All statistical tests were performed using one-way ANOVA or parametric *t*-test, **P < 0.01, ***P < 0.001, ****P < 0.0001 to siAllStar unless indicated.

When cancer cells experience non-lethal doses of chemotherapeutics, as encountered when the drug concentration declines during treatment (Gewirtz, 1999), the cells can enter a state of therapy-induced senescence (Ewald et al., 2010). Clinically, there is evidence that therapy-induced senescence is associated with good prognosis, however there are also indications that a proliferative subpopulation can emerge with adverse effects and cancer relapse (Demaria et al., 2017). p21 is a master regulator of therapy-induced senescence, but emerging evidence also demonstrates that p21 can induce cell proliferation after chemotherapy (Abbas and Dutta, 2009; Cazzalini et al., 2010). Interestingly, the heterogeneous expression of p21 in an isogenic population of cancer cells was recently linked to cell fate decisions after non-lethal doses of chemotherapeutic treatment (Hsu et al., 2019). In that study, the authors demonstrated that the cell cycle phase and the expression level of p21 would determine if a cancer cell becomes senescent or begins to proliferate after recovering from chemotherapy. In brief, their data demonstrated that cells in G1-phase, expressing intermediate levels of p21, would become proliferative after drug recovery, but only if the cells maintained low DNA damage during treatment (Hsu et al., 2019). On the other hand, cells with too low or too high p21 levels would lead to therapy-induced senescence. More importantly, the proliferation fate of the cells also relied on the presence of intermediate p21 levels prior or during the drug treatment (Hsu et al., 2019). This “p21-goldilocks zone,” as the authors called the scenario, is reminiscent to the loss of STRIP1 in MDA-MB-231 cells, where cells are arrested in G1-phase with induced levels of p21. The similarity was further emphasized by the observation that loss of STRIP1 also reduces basal levels of DNA damage response, as demonstrated by immunoblotting and immunofluorescence analyses of γH2AX (Figures 4A,B). Gene set enrichment analysis (GSEA) further confirmed the downregulation of genes involved in G0-G1 DNA damage checkpoint (Supplementary Figure 1F).

These observations prompted us to investigate if loss of STRIP1 and the subsequent activation of MST3&4 would influence cell fate decision after treatment with chemotherapy. We decided to test doxorubicin as it is one of the most commonly used chemotherapeutics in the clinic. We began by treating siRNA-depleted cells with high dose of doxorubicin (1 μM) for only 6 h (experimental set-up, Figure 4C). Single cell immunofluorescence analysis revealed that treatment induced high p21 expression in ~20% of control cells, while the numbers increased to ~60% in STRIP1-depleted cells (Figure 4D). Once again, the p21 levels could be reverted to that of control cells by co-depleting MST3&4 (Figure 4D).

The “p21-Goldilocks zone” dictates that the p21/γH2AX ratio in individual cells has to be high, if cells have to become proliferative rather than senescent (Hsu et al., 2019). We therefore quantified the p21/γH2AX ratio by immunofluorescence analysis and observed that loss of STRIP1 increased the p21/γH2AX ratio within single cells (Figure 4E). Importantly the amplified p21/γH2AX ratio could be reverted to control levels after depleting MST3&4 (Figure 4E). Consistently, loss of STRIP1 also increased the p21/γH2AX ratio in single cells treated with a second chemotherapeutic, cisplatin (Figure 4F). The findings indicate that hyper-activated MST3&4 can promote a “cell state”, matching the “p21-Goldilocks zone,” which may facilitate cell proliferation rather than senescence, if treated with sub-lethal dose of chemotherapeutics.

### STRIP1 Regulates Proliferation-Senescence Cell Fate After Chemotherapy

**Figure 5:**
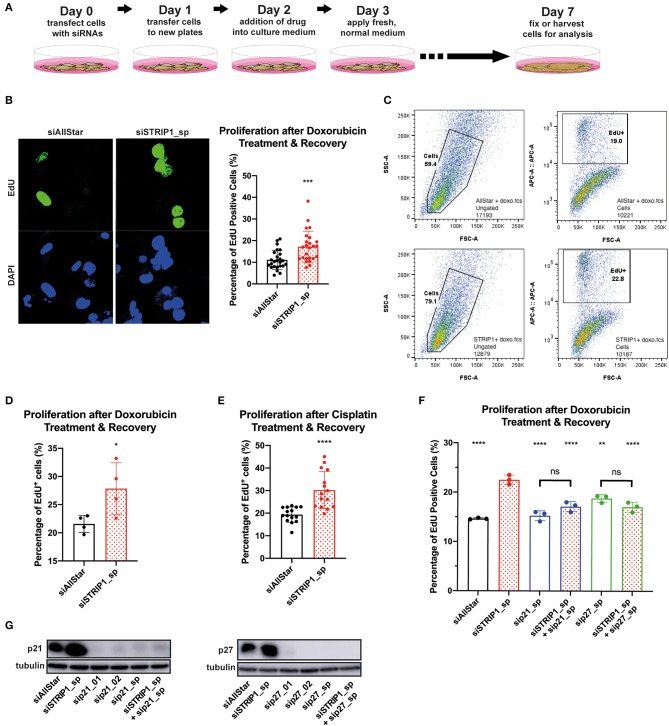
STRIP1 regulates proliferation-senescence cell fate after chemotherapy. **(A)** Experimental set-up of the drug treatment experiment. **(B–F)** siRNA-depleted MDA-MB-231 cells were treated with low dose chemotherapy for 24 h and allowed to recover for a further 96 h. EdU was added to cells in culture 2 h prior to fixation/harvesting and analyzed for proliferation status. **(B)** Immunofluorescence quantification and representative images show increased EdU^+^ cells after loss of STRIP1 with doxorubicin treatment. **(C)** Gating strategy for EdU proliferation flow cytometry analysis. Side scatter area and forward scatter area were used to obtain the whole cell population (Cells). The population is then analyzed according to EdU intensity (APC conjugated) against forward scatter area to identify proliferative cells (EdU+). **(D)** Flow cytometry quantification shows increased EdU^+^ cells after loss of STRIP1 with low dose doxorubicin treatment and subsequent recovery. **(E)** Immunofluorescence quantification of EdU^+^ cells shows increased proliferation after loss of STRIP1 with low dose cisplatin treatment and recovery. **(F)** Flow cytometry quantification of STRIP1 and p21 or p27 co-depleted cells with decreased EdU+ populations compared to STRIP1-depleted cells. Statistical analysis was compared to siSTRIP1 unless indicated. All immunofluorescence and flow cytometry analyses were conducted at least three independent times. **(G)** p21 and p27 protein knockdown efficiencies of siRNA-depleted MDA-MB-231 cells were demonstrated by immunoblotting. All statistical tests were performed using parametric *t*-test or one-way ANOVA, *P < 0.05, **P < 0.01, ***P < 0.001, ****P < 0.0001 to siAllStar unless indicated.

To answer that hypothesis, we treated siRNA-depleted cells with low dose doxorubicin (50 nM) for 24 h and then allowed the cells to recover for 4 days without the presence of doxorubicin (experimental set-up, Figure 5A). This dosage rarely induces apoptosis but is still sufficient to be clinically relevant (Gewirtz, 1999; Hsu et al., 2019). Accordingly, the majority of cells will enter therapy-induced senescence, while a minor subpopulation may re-enter the cell cycle and start to proliferate (Hsu et al., 2019). To quantify the magnitude of cells that re-entered the cell cycle during recovery-phase, we treated the cells for 2 h with ethynyl-20-deoxyuridine (EdU). We then analyzed single cells for EdU incorporation into newly synthesized DNA, by immunofluorescence and flow cytometry. In both analyses, the loss of STRIP1 significantly increased the numbers of cells entering the cell cycle, as compared to control cells (Figures 5B–D). Importantly, these observations could also be reproduced using non-lethal doses of cisplatin (Figure 5E). As further affirmation of p21's role in a proliferative cell fate, we co-depleted STRIP1 with p21 and subjected the cells to the same low dosage chemotherapy. Indeed, p21-depletion rescued the increased proliferation of STRIP1-depleted cells (Figure 5F). Interestingly, we were also able to rescue the proliferative cell fate by co-depleting p27 in STRIP1-depleted cells (Figure 5F). Immunoblotting analysis validated that all siRNAs successfully depleted the p21 and p27 proteins (Figure 5G). In summary, these experiments demonstrate that STRIP1-regulation of p21 and p27 influences cell fate decisions after non-lethal doses of chemotherapy.

## Discussion

The STRIPAK complex has, in recent years, been linked to the progression of cancer (Wong et al., 2014; Zhang et al., 2014; Madsen et al., 2015; Huang et al., 2017). In breast cancer, the complex regulates the mode of migration of cancer cells and consequently, the ability of cells to metastasize (Madsen et al., 2015). The migration mode relies on the activation state of the two MST3&4 kinases. Hyper-activated MST3&4 couples the actomyosin network to the plasma membrane, while hypo-activated MST3&4 links actomyosin to integrins via focal adhesions. This determines whether cells move in an amoeboid or mesenchymal way, respectively (Madsen et al., 2015). In this study, we demonstrate that loss of STRIP1 in breast cancer cells also induces p21 and p27 expression in a MST3&4 dependent manner. As a consequence, the cells are arrested in the G1-phase causing a reduction in cell proliferation and tumor growth. Surprisingly, the induction of p21 was limited to a subpopulation of cells, which also exhibited low levels of DNA damage response. This phenotype of cells, arrested in G1-phase with increasing p21 and low γH2AX expression (high p21/γH2AX ratio), is reminiscent to the “Goldilocks zone” observed in lung cancer cells recovering from non-lethal dosage of chemotherapeutics (Hsu et al., 2019). These observations made us test if loss of STRIP1 would promote a “cell population” in favor of becoming proliferative rather than senescent after treatment with sub-lethal doses of chemotherapy. To our big surprise this was indeed the case, loss of STRIP1 promoted the recovery of breast cancer cells from both doxorubicin and cisplatin treatment. It is important to state that we did not examine in detail how the p21/γH2AX ratio was regulated in the heterogeneous cell population. We can conclude that the p21 expression is dependent on MST3&4, but we do not know if the levels of γH2AX also relies on these kinases.

The fact that loss of STRIP1 suppresses DNA damage response may actually have detrimental consequences, as it may result in an escaped population of cell with high levels of DNA damage and genomic instability. This has indeed been demonstrated in osteosarcoma cells having prolonged p53-independent expression of p21 (Galanos et al., 2016). The inverse correlation between p21 and γH2AX, with corresponding DNA instability, has also been documented in breast cancer cells (Yaglom et al., 2014).

In conclusion, our observations demonstrate a conflicting function of STRIP1 in regulating proliferation of breast cancer; low levels of STRIP1 suppress proliferation of untreated cells while inducing proliferation of cells recovering from non-lethal doses of chemotherapy. These observations can be justified by the regulation of p21 and p27, which has been shown to exhibit both tumor-suppressive and tumor-promoting functions (Abbas and Dutta, 2009).

From a speculative perspective, these observations suggest that low levels of STRIP1 may correlate with good prognosis in untreated patients, due to lower tumor growth. On the contrary, low STRIP1 levels would have a poor prognosis in patients receiving chemotherapy, due to recovery and recurrence of treated cancer cells. To investigate this idea, we took advantage of a microarray datasets of breast cancer patients using the online resource; http://kmplot.com/analysis (Gyorffy et al., 2010). First, we looked at breast cancer patients, which had never received any treatment. As the number of patients were very low, this analysis was not statistically valid, but nevertheless supported the hypothesis that low levels of STRIP1 may be beneficial (Supplementary Figure 2A). We then focused our attention to patients that had received systematic treatment. In stark contrast to untreated patients, low STRIP1 levels correlated with poor prognosis as hypothesized (Supplementary Figure 2B). We then restricted the analysis to patients that had received chemotherapy (in conjunction with endocrine therapy). The analysis also supported low levels of STRIP1 to correlate with poor prognosis (Supplementary Figure 2C). Although these Kaplan Meier analyses are inconclusive due to low patient numbers, they do support the notion that STRIP1 may play an important role in breast cancer. The assumption would however need further validation in animal models and clinical specimens.

From a mechanistic perspective, we demonstrate that loss of STRIP1 impedes the cell cycle progression and proliferation of breast cancer cells by inducing expression of p21 and p27, two bonafide CDK inhibitors and G1 checkpoint regulators. These observations are emphasized by the limited transcription of E2F-target genes, needed for the progression into the S-phase (Bertoli et al., 2013). The loss of STRIP1 has formerly been linked to the activation of the Hippo-kinases and the suppression of YAP/TAZ-induced proliferation (Tang et al., 2019). However, in our case we did not observe changes in Hippo-YAP/TAZ signaling, implying another mechanism of cell cycle regulation. Contrary to this, we demonstrate that the expression of p21 and p27 are strictly dependent on the stimulation of MST3&4. Although we did not look into the molecular mechanism, MST3 has been shown to regulate p21 phosphorylation and stability through the activation of NDR1/2 kinases (Cornils et al., 2011). Indeed, the phosphorylation state of p21 is an important regulator of its function, as it controls the stability and the cellular localization, as well as its direct binding to PCNA (Karimian et al., 2016). When p21 is phosphorylated, for example by AKT, the p21-PCNA bond is disrupted and the PCNA protein is now free to form a complex with the DNA polymerase δ holoenzyme to promote DNA replication (Karimian et al., 2016). Our data clearly demonstrates that loss of STRIP1 reduces AKT phosphorylation in an MST3&4 dependent manner. In yeast, the *Strip1* homolog was shown to antagonize the mTOR complex 2, thus affecting AKT activity (Pracheil et al., 2012). Thus, it seems plausible that STRIP1-MST3&4 may regulate cell proliferation through AKT regulation. An alternative explanation is that STRIP1-MST3&4 regulate p21 and p27 stability by regulating its phosphorylation state. Although we did not examine the phosphorylation of p21 and p27, we never saw any difference in their subcellular localization; the proteins were always localized to the nucleus of MDA-MB-231 cells. Hence, the link between STRIP1-MST3&4 and the phosphorylation state and stability of p21 and p27 awaits further examinations. In this regard, it is important to state that the phosphorylation of p21 has been shown to have both CDK inhibitory functions and cell proliferative promoting functions depending on the cellular context.

In conclusion, our findings suggest that STRIP1 antagonizes the two MST3&4 kinases in breast cancer cells. This may suppress tumor growth as shown in the study, but unfortunately also induce the dissemination of metastasis as previously shown (Madsen et al., 2015). On top of that, hyper-activated MST3&4 promote a subpopulation of breast cancer cells having low DNA damage response with the ability to recover from low dosage of chemotherapy.

## Data Availability Statement

The RNA-sequencing data generated in this study have been deposited in the GEO database under accession GSE145618.

## Ethics Statement

The animal study was reviewed and approved by Malmø - Lund ethical experimental animal committee. Written informed consent was obtained from the individual(s) for the publication of any potentially identifiable images or data included in this article.

## Author Contributions

CR-C, MD, LS, EE, PK, SW, DL, HA, and AG-M carried out the experiments. CM carried out the Kaplan–Meier and mRNA co-expression analyses and designed the project. AG-M and CM supervised the project. CR-C and CM wrote the article. All authors discussed the results and commented on the manuscript text.

### Conflict of Interest

The authors declare that the research was conducted in the absence of any commercial or financial relationships that could be construed as a potential conflict of interest.
