# Supplementary figures and images for: The STRIPAK Complex Regulates Response to Chemotherapy Through p21 and p27
Source: Front Cell Dev Biol. 2020 Mar 17;8:146. doi: 10.3389/fcell.2020.00146 (PMC7089963; doi:10.3389/fcell.2020.00146)

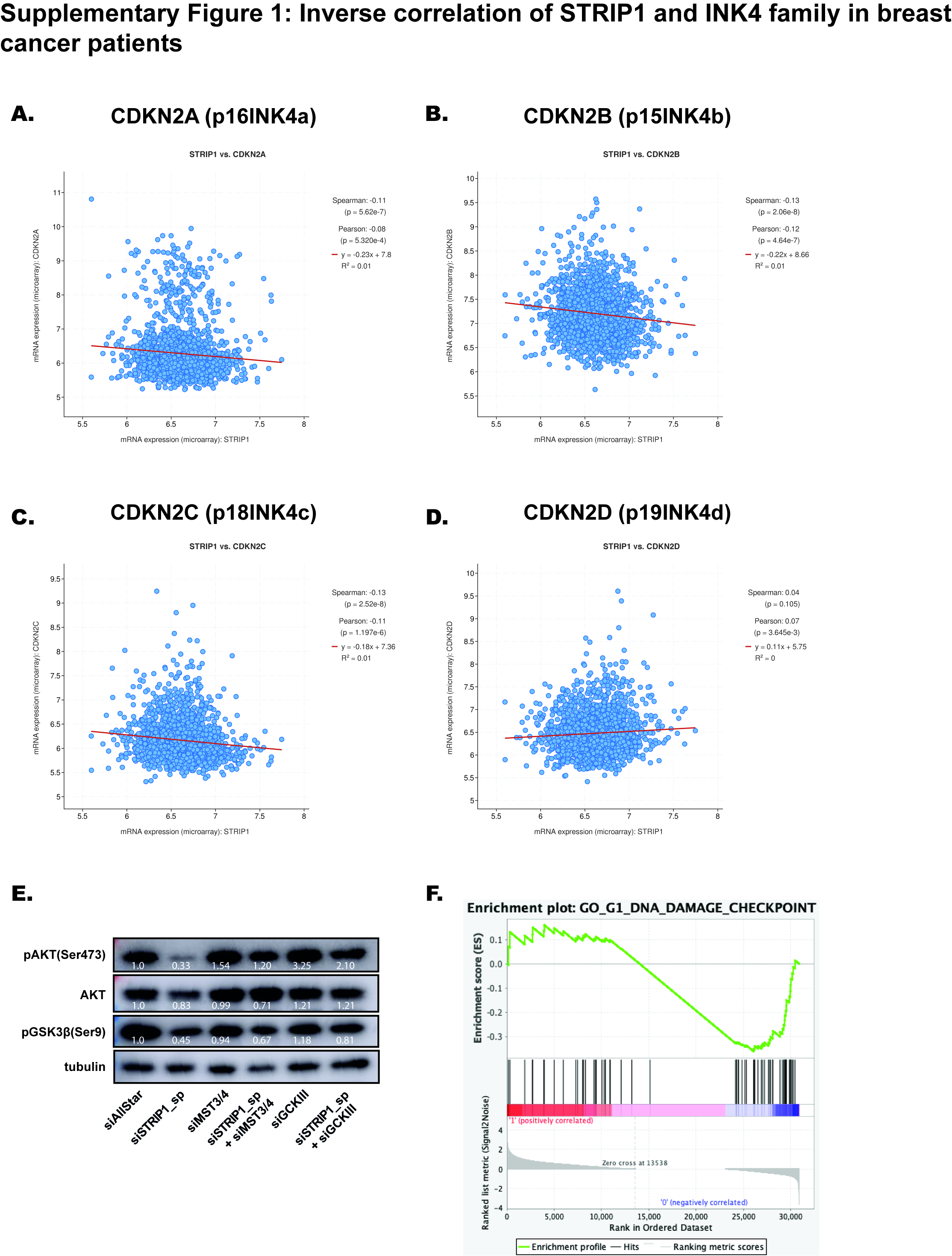

Supplement: Supplementary file 1 [file Image_1.TIF]

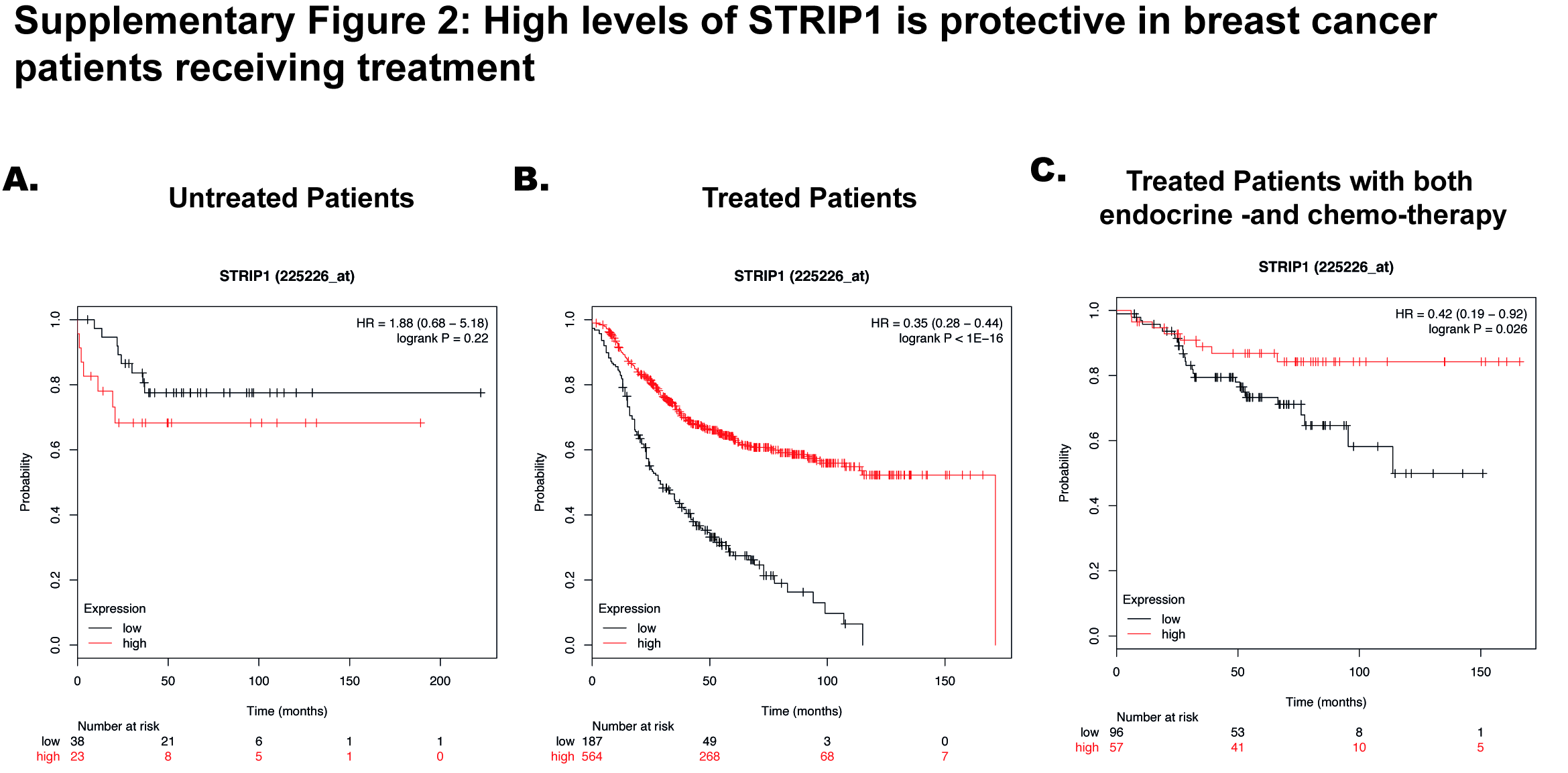

Supplement: Supplementary file 2 [file Image_2.TIF]
